# Genomic characterization of Clostridioides (Clostridium) difficile in Cambodia

**DOI:** 10.1099/mgen.0.001881

**Published:** 2026-11-02

**Authors:** Lengsea Eng, Daniel R. Knight, Deirdre A. Collins, Natasza Hain-Saunders, Kefyalew Addis Alene, Archie C.A. Clements, Thomas V. Riley

**Affiliations:** 1 School of Population Health, Faculty of Health Sciences, Curtin University, Perth, Western Australia, Australia; 2 Department of Laboratories, Calmette Hospital, Phnom Penh, Cambodia; 3 PathWest Laboratory Medicine, Perth, Western Australia, Australia; 4 School of Biomedical Sciences, The University of Western Australia, Perth, Western Australia, Australia; 5 School of Medical and Health Sciences, Edith Cowan University, Joondalup, Western Australia, Australia; 6 Geospatial and Tuberculosis Research, The Kids Research Institute Australia, Nedlands, Western Australia, Australia

**Keywords:** antimicrobial resistance (AMR), Cambodia, *Clostridioides difficile*, core-genome SNP (cgSNP), ribotype, sequence type

## Abstract

**Background.** Genomic studies can provide insights into genetic determinants of virulence, antimicrobial resistance (AMR) and transmission pathways of *Clostridioides difficile*. Here, we characterized the core genome of *C. difficile* strains belonging to the five most common ribotypes (RTs) found in Cambodia.

**Methods.** Whole-genome sequencing was conducted on 48 toxigenic *C. difficile* strains belonging to RTs 017, 012, 046, 014/020 and 056, recovered in 3 previous epidemiological studies in Cambodia between 2022 and 2023. Toxin and AMR genes were detected, and MLST was determined. Comparative genomic analysis was conducted within strains isolated in Cambodia and between strains from Cambodia and Thailand.

**Results.** *C. difficile* ST2/RT 014/020 was the most genetically diverse [14–2,563 core-genome SNPs (cgSNPs)], whilst ST37/RT 017 was the most clonal (0–32 cgSNPs). Clonal strains (defined as sharing ≤2 cgSNPs) were observed within ST34/RT 056, ST37/RT 017 and ST54/RT 012 groups. One strain of ST37/RT 017 from Cambodia differed by eight cgSNPs from a strain from Thailand isolated in 2018. Resistance genes *vanZ1* was the most prevalent, followed by *tet(M)*, *vanR-Cd*, *vanS-Cd*, *vanG-Cd*, *vanT-Cd*, *blaCDD-2*, *erm(B)*, *blaCDD-1* and *aph(2’)-Ih*. Genotypic multidrug resistance, genotype associated with the phenotype, was found in 100% of ST37/RT 017 strains and 67% of other strains.

**Conclusions.** The transmission of *C. difficile* in Cambodia occurred not only among genetically distinct strains but also clonal strains. Besides sources within the country, *C. difficile* could have also originated from neighbouring countries such as Thailand. A high number of AMR determinants in most strains indicates the necessity for strict AMR surveillance in the country.

**Impact Statement**

Whole-genome sequencing (WGS) of 48 toxigenic *Clostridioides difficile* strains from Cambodia revealed silent transmission of clonal strains and suggested that sources and reservoirs of *C. difficile* in Cambodia were both from within and outside the country. WGS also indicated high numbers of AMR genes harboured by *C. difficile* in this country.

## Data Summary

The whole-genome sequence data generated in this study were submitted to GenBank NCBI under BioProject PRJNA 1358371, with accession numbers SRR37271971–SRR37272018. A summary of isolates, sequence data and comparative sequence matrix are available in the Supplementary Documents.

## Introduction

As one of the most common causes of hospital-associated and community-associated infections, *Clostridioides* (*Clostridium*) *difficile* has been extensively studied in relation to the epidemiology of infections and antimicrobial resistance (AMR). Whole-genome sequencing (WGS) of the *C. difficile* genome has revealed important information about virulence factors, including toxin A, toxin B and binary toxins, AMR genes and genetic identity/variation of different circulating strains within hospitals and the community [1]. Variations in the genome via mutations or acquisitions of new genetic material, e.g. plasmids, contribute to the emergence/outbreaks of hypervirulent strains [2], such as sequence type (ST) 1/PCR ribotype (RT) 027 in North America and Europe [3], and ST37/RT 017 worldwide [4].

Several studies in Asia found many diverse novel RTs, mostly belonging to evolutionary clade 4, including ST37/RT 017 as one of the most predominant genotypes [5–8]. Imwattana *et al*. studied 282 *C*. *difficile* ST37/RT 017 strains finding 2 lineages circulating globally, 1 epidemic and 1 non-epidemic [4]. The epidemic strain of *C. difficile* ST37/RT 017 possessed an *ermB* gene, the appearance of which was related to the introduction of clindamycin into clinical practice. However, these studies did not include strains from Cambodia.

A study in the Asia-Pacific region indicated a variation in genotype predominance of circulating *C. difficile* in each country; for example, *C. difficile* RT 017 was predominant in Thailand, Vietnam and Singapore; RT 014/020 was predominant in Australia, and RT 018 was predominant in Japan [5]. The study did not determine if these predominant strains were clonal or genetically distinct, suggesting a need to further explore the genetic identity of these strains or to identify possible outbreaks. Epidemiological studies on *C. difficile* in Cambodia have been conducted recently in hospitalized adults [7], hospitalized children [9], adolescents in the community and children visiting an outpatient department (OPD) [8]. Antimicrobial susceptibility testing (AST) was conducted on 192 isolates from these 3 studies [10].

The current study aimed to characterize the core genome and AMR repertoire of toxigenic *C. difficile* strains comprising the five most common RTs of *C. difficile* circulating in Cambodia.

## Methods

### Strain collection

The collection of 48 toxigenic strains from previous studies included 17 isolates from hospitalized adults [7], 11 isolates from hospitalized children [9], 5 isolates from adolescents in the community and 15 isolates from children visiting an OPD [8] (Table 1). The number of isolates included varied based on the number of toxigenic *C. difficile* recovered in those studies. All strains were isolated from samples collected between January 2022 and March 2023. The three studies were cross-sectional; thus, only one strain was isolated from one patient.

**Table 1.** Number of *C. difficile* isolates by population studied

Ribotype	Hospitalized adults	Hospitalized children	OPD children	Community adults	Total
017	4	1	8	1	14
012	2	7	2	3	14
046	5	0	2	0	7
014/020	3	3	0	0	6
056	3	0	3	1	7
**Total**	**17**	**11**	**15**	**5**	**48**

### WGS and bioinformatics analysis

Genomic DNA (gDNA) was extracted from a 48 h blood agar anaerobic culture of *C. difficile* using a DSP DNA purification kit on the QIAGEN QIASymphony SP platform (QIAGEN, Hilden, Germany). DNA concentrations were estimated using Qubit spectrophotometry. *C. difficile* isolates were sequenced using an MGI DNBSEQ-G99 sequencer (MGI, Shenzhen, China) to an average read depth of 230×. The extracted gDNA was prepared for sequencing with the Illumina^®^ DNA Prep Library Kit (Illumina, San Diego, CA, USA) at a modified reagent volume ratio of 0.5. A summary of sequenced isolates and associated metadata is provided in Supplementary materials S1 and S2 (available in the online Supplementary Material). Sequence data are available under NCBI BioProject PRJNA1358371 (accessions SRR37271971–SRR37272018, Supplementary material S2).

Species identification was performed with Kraken2 v2.0.8 (https://github.com/Derrick Wood/kraken2). Whole-genome sequences were characterized for toxin genes using Abricate v1.0.1 (https://github.com/tseemann/abricate) and multi-locus sequence type (MLST) using mlst v2.19.0 (https://github.com/tseemann/mlst) and the scheme of Griffiths *et al*. [11]. The presence of AMR genes (horizontally acquired and chromosomal) was determined using Abricate v1.0.1 with the virulence factor database (vfdb) 20-06-2026. Mutations within the quinolone resistance-determining region of DNA gyrase genes *gyrA* and *gyrB* were determined using a custom database of DNA gyrase allele sequences from PubMLST (https://pubmlst.org/organisms/clostridioides-difficile) compiled within Abricate v1.0.1.

Genomic clustering was based on pairwise core-genome SNP (cgSNP) distances from reference-free alignment of variant split *k*-mers across whole genomes using SKA v1.0 (https://github.com/simonrharris/SKA). A maximum likelihood cgSNP tree was generated using IQtree v1.6.2 (https://iqtree.github.io/) with the General Time Reversible substitution model and curated in iTol v6.5.2 (https://itol.embl.de/). Single-linkage clustering was performed, and a threshold of ≤2 SNPs was used to define a genomic cluster [12]. Without considering the molecular evolutionary complexities within the dormant spore phase, the criterion of ≤2 cgSNP difference was applied for clonal strains and ≤10 cgSNP difference for closely related strains [12].

Pairwise cgSNP analysis was also performed between strains from Cambodia and Thailand, using strains isolated from Thailand between 2014 and 2018 with available genomic sequence data in our laboratory: 1 isolate of ST54 and 50 isolates of ST37 (Supplementary material S3). All strains from Thailand were from human cases of *C. difficile* infection (CDI). Strains from another neighbouring country, Vietnam, were not included in the study due to the unavailability of genomic sequence data.

### Antimicrobial susceptibility

Phenotypic AST was performed using agar dilution as previously described by Eng *et al*. [10]. MIC was determined following the Clinical and Laboratory Standards Institute (CLSI) guidelines for a panel of ten antimicrobial agents, as described by Eng *et al*. [10]. *C. difficile* ATCC 700057, *Bacteroides fragilis* ATCC 25285, *Eubacterium lentum* ATCC 43055 and *Bacteroides thetaiotaomicron* ATCC 29741 were used as quality control strains. The MIC results for the 48 isolates are given in Supplementary material S4. Phenotypic multidrug resistance (MDR) was defined as phenotypic resistance to at least three antimicrobial classes, and genotypic MDR was determined as possessing AMR genes conferring resistance to at least three antimicrobial classes, excluding intrinsic resistance [13].

## Results

### Genome characteristics and multi-locus sequence type versus ribotype

The genome sizes of the 48 strains differed slightly between strains, 4.00–4.42 Mbp (Table 2). Forty-six of the 48 toxigenic strains of *C. difficile*, including RTs 017, 012, 046, 014/020 and 056, were identified by *in silico* MLST as belonging to 5 STs, including 37, 54, 35, 2 and 34, respectively (Table 2). There was a good correlation between RTs and STs except for two isolates of RTs 017 and 056 that were novel STs (Fig. 1). The sequence data for these two isolates were submitted to PubMLST and confirmed as ST1246 and ST1247, respectively. Except for the 2 novel STs, 33 out of 46 (72%) isolates were classified as belonging to evolutionary clade 1 and 13 out of 46 (28%) to evolutionary clade 4.

**Table 2.** Genome characteristics of the 48 toxigenic *C. difficile*

Ribotype	**ST**	N isolates	Evolutionary clade	Genome size (Mbp)
014/020	ST2	6 (12.5%)	1	4.02–4.25
56	ST34	6 (12.5%)	1	4.00–4.18
046	ST35	7 (14.6%)	1	4.22–4.42
017	ST37	13 (27.0%)	4	4.13–4.26
012	ST54	14 (29.2%)	1	4.15–4.27
056, 017	Novel	2 (4.2%)	–	4.03–4.08
**Total**		**48**		

**Fig. 1.**
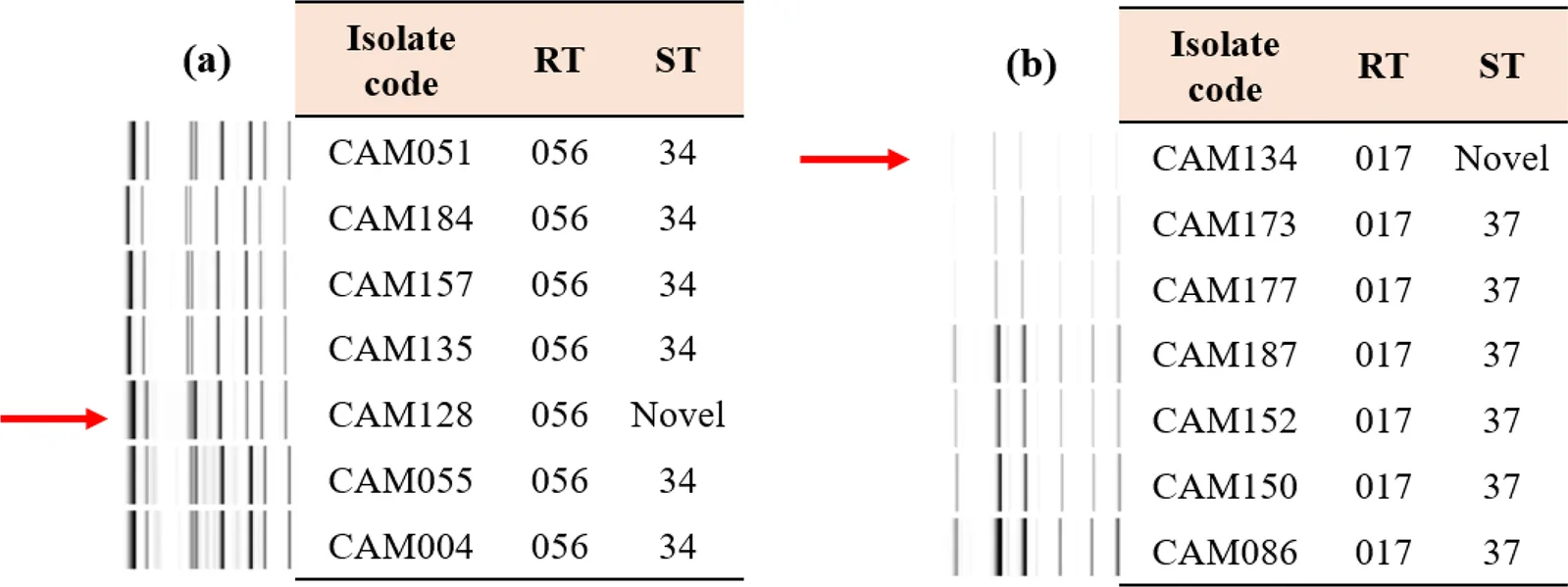
Comparison of ribotyping banding pattern of the novel *C. difficile* STs among RTs 056 (a) and 017 (b).

### cgSNP analysis for strain relatedness within the cohort and between the cohorts

The number of pairwise cgSNP differences within ST ranged from 14 to 2,563 cgSNPs for ST2, the largest difference, and 0–32 cgSNPs for ST37, the smallest difference (Table 3), excluding the 2 novel STs.

**Table 3.** cgSNP ranges within STs of *C. difficile*

ST/RT	No. of isolates	cgSNP range
2/014/020	6	14–2,563
34/056	6	1–57
35/046	7	8–348
37/017	13	0–32
54/012	14	0–135
**Total**	**46**	

Clonal strains (≤2 cgSNP difference) accounted for an overall 24% (11/46) of the total STs, or at least 15% of ST37 and at most 50% of ST54. Genetically distinct strains occurred in an overall 41% (19/46) of the total, or between 14% in ST54, 71% in ST35 strains and 100% in ST2.

Clonal pairs of strains (≤2 cgSNP difference) were observed within STs 34, 37 and 54, isolated from samples collected between 1 and 10 days apart, within study participant groups: hospitalized adults, hospitalized children and adolescents in the community (Fig. 2). Some of the paired study participants were residents of the same region in the country, and some of them were residents in different regions. There was no pair of clonal strains identified from both healthcare and community settings.

**Fig. 2.**
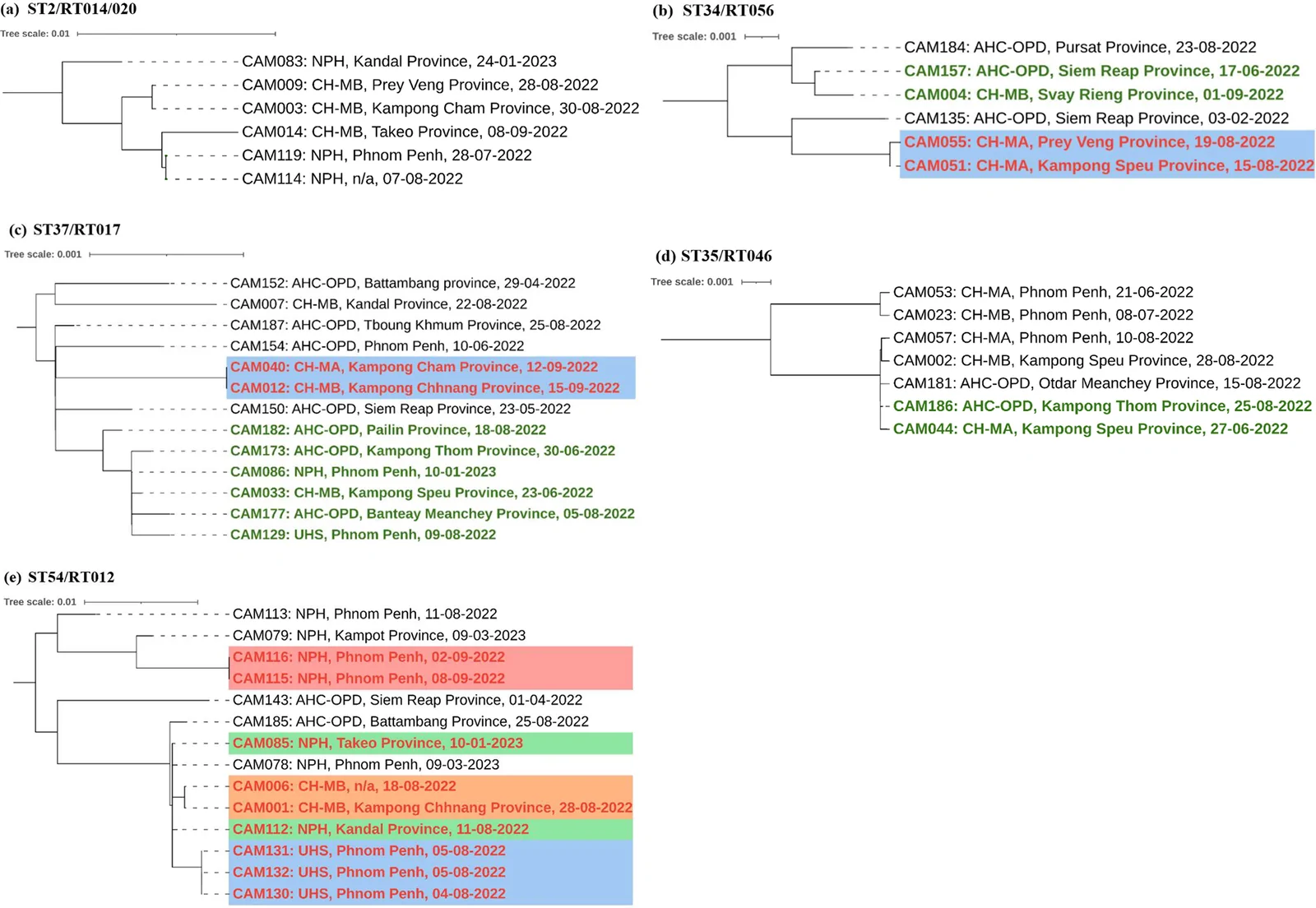
Maximum-likelihood phylogeny based on cgSNP. Taxon label includes isolate ID: institute, host origin and isolation date. Isolates written in red differed by ≤2 cgSNPs, those in green differed by ≤10 cgSNPs and those in black differed by >10 cgSNPs. Isolates were clustered with a threshold of ≤2 cgSNPs. (a) Six isolates of ST2/RT014/020, with no cluster; (b) 6 isolates of ST34/RT056, with 1 cluster; (c) 13 isolates of ST37/RT 017, with 1 cluster; (d) 7 isolates of ST35/RT 046, with no cluster; and (e) 14 isolates of ST54/RT 012, with 4 clusters identified. Different highlight colours indicate different clusters. NPH, National Paediatric Hospital; CH-MB, Calmette Hospital – Medicine B ward; AHC-OPD, Angkor Hospital for Children – outpatient department; CH-MA, Calmette Hospital – Medicine A ward; UHS, University of Health Sciences.

Closely related *C. difficile* strains (≤10 cgSNP difference) were found within and between study participant groups, within ST34/RT 056, ST37/RT 017, ST35/RT 046 and ST54/RT 012. Those strains were isolated from samples collected at least 10 days, and up to 7 months, apart (Fig. 2). Except for those with unknown residency, all the paired study participants were residents of different regions. Strains with >10 cgSNP differences were also observed within and between study participant groups. Some of them were residents of the same region, and some were from different regions.

### cgSNP analysis of strain relatedness between strains from Cambodia and Thailand

With only one strain of *C. difficile* ST54 (code B14-21) isolated in 2014 with available genomic sequence data, the comparative genomic analysis with the 14 strains of ST54 isolated in Cambodia found no clonal relatedness (Supplementary material S5). The closest relatedness was 27 cgSNP differences with 1 strain from Cambodia (code CAM085). Between the 13 strains of ST37 from Cambodia and 50 strains of ST37 isolated in Thailand, there was 1 strain from Thailand (code MAR272) isolated in 2018, which differed by 8 cgSNPs from a strain from Cambodia (code CAM187), and by 12–22 cgSNPs from all other 12 strains of ST37 in Cambodia (Supplementary material S6).

### AMR and the difference between phenotype and genotype

No *C. difficile* isolate was resistant phenotypically or genotypically to metronidazole, fidaxomicin or rifaximin. Among the 48 toxigenic isolates, at least 2 and up to 11 AMR determinants, including acquired resistance genes and genes with mutations, were detected in each strain.

There were some discrepancies between phenotypic and genotypic resistance for vancomycin, clindamycin, meropenem, tetracycline and moxifloxacin (Table 4). All isolates were phenotypically susceptible to vancomycin, while different resistant genes, including *vanR-Cd*, *vanS-Cd*, *vanG-Cd* or *vanT-Cd*, were identified in 68.8% (33/48) and *vanZ1* in 79.2% (Table 4). None of the strains was susceptible to clindamycin, 12.5% (06/48) showed intermediate susceptibility and 87.5% (42/48) were resistant. None of the isolates with intermediate susceptibility to clindamycin harboured *erm(B)*. In the strains phenotypically resistant to clindamycin, *erm(B)* was detected in only 47.6% (20/42). For moxifloxacin, the presence of *gyrA* p.T82I was 100% concordant with phenotypic resistance, while *gyrB* p.S366A had 0% concordance (Supplementary material S7).

**Table 4.** AMR, phenotype and genotype, of the 48 toxigenic *C. difficile*

Antimicrobial	Phenotypic resistance		Genotypic resistance			
	MIC range (mg/l)	No. of isolates (%)	Acquired resistance genes		Genes of mutation	
Vancomycin	0.5–2	0/48 (0%)	*vanR-Cd*	33/48 (68.8%)	–	–
			*vanS-Cd*	33/48 (68.8%)	–	–
			*vanG-Cd*	33/48 (68.8%)	–	–
			*vanT-Cd*	33/48 (68.8%)	–	–
			*vanZ1*	38/48 (79.2%)	–	–
Metronidazole	0.12–0.25	0/48 (0%)	–	–	–	–
Fidaxomicin	0.03–0.12	0/48 (0%)	–	–	–	–
Rifaximin	0.008–0.03	0/48 (0%)	–	–	–	–
Clindamycin	4–>64	42/48 (87.5%)	*erm(B*)	20/48 (41.7%)	–	–
Erythromycin	0.5–>512	–			–	–
Meropenem	2–8	0/48 (0%)	*blaCDD-1*	20/48 (41.7%)	–	–
			*blaCDD-2*	28/48 (58.3%)	–	–
Amoxicillin/clavulanic acid	0.5–2	0/48 (0%)	–	–	–	–
Tetracycline	0.25–64	32/48 (67%)	*tet(M)*	34/48 (70.8%)	–	–
Moxifloxacin	2–32	03/48 (6.3%)	–	–	*gyrA* p.T82I	03/48 (6.3%)
			–	–	*gyrB* p.S366A	15/48 (31.3%)
Chloramphenicol	Not tested	n/a	*catD*	02/48 (4.2%)	–	–
Aminoglycosides	Not tested	n/a	*aadE*	02/48 (4.2%)	–	–
			*aph(2’)-Ih*	16/48 (33.3%)	–	–

Phenotypic MDR occurred in 2 out of 7 strains within ST35/RT 046 (Supplementary material S7), while genotypic MDR occurred in 37 out of 48 strains: 14 out of 14 strains within ST54/RT 012, 13 out of 13 strains within ST37/RT 017, 7 out of 7 strains within ST35/RT 046, 1 out of 6 strains within ST2/RT 014/020 and 0 out of 6 strains within ST35/RT 056 and the 2 novel STs.

By clade, none of the clade 4 *C*. *difficile* ST37/RT 017 strains contained *erm(B)*, *aph(2’)-Ih*, *blaCDD1* or *gyrA* p.T82I, but all of them had *tet(M)*, *vanZ1* and *gyrB* p.S366A (Supplementary material S7). While genotypic MDR occurred in all 13 strains in clade 4, it occurred in 67% (22/33) of strains in other clades. None of the ST2/RT 014/20 strains contained *erm(B)*, *tet(M)*, *aph(2’)*, *aadE*, *catD* or mutation in *gyrB* (p.S366A), but all were resistant to clindamycin (Supplementary material S7).

Chloramphenicol and aminoglycosides were not included in AST [10]; however, resistance determinants, including *catD* and *aadE*, were detected in two out of seven strains within ST35/RT 046 (Supplementary material S7)

## Discussion

This is the first study of the genetic characterization of *C. difficile* strains from Cambodia using WGS. All *C. difficile* strains in this study were from individuals both with and without diarrhoea who were not necessarily diagnosed with CDI disease. Knowing that *C. difficile* ST37/RT 017, belonging to evolutionary clade 4, is the most predominant group of *C. difficile* strains in Asia [14], an isolate previously identified as RT 017 (Fig. 1) was identified as a novel ST in the current study, suggesting that more strains similar to *C. difficile* RT 017 in clade 4 in this country are likely to be found.

### Spread of *C. difficile* within and between settings

Based on cgSNP differences, several clonal pairs of strains were identified within the same healthcare setting (Fig. 2), within and between wards, as well as between individuals in the same cohort of adolescents in the community. Some of them were from the same region of residence, and some were from a different region. These findings suggest that transmission of clonal strains does occur either in healthcare settings or within the community. Interestingly, clonal strain transmission in the current study occurred only within ST34/RT 056, ST37/RT 017 and ST54/RT 012 strains isolated within no more than 10 days of each other (Fig. 2). Knowing that these strains were from patients with *C. difficile* colonization rather than symptomatic infection [7–9] strongly suggests ongoing silent transmission.

Closely related strains within the ST34/RT 056, ST37/RT 017, ST35/RT 046 and ST54/RT 012 groups (Fig. 2), indicating strains sharing a recent genomic relationship [12], were found in study participants within and in different healthcare facilities and from different regions of residence. It can be hypothesized that multiple silent transmissions occur in the community, or from one region to another within the country and, plausibly, between neighbouring countries like Thailand and Vietnam, which harbour similar strains [6, 15]. Though clonal transmission and the transmission of *C. difficile* of shared ancestry were identified, the circulation of genetically distinct *C. difficile* also occurred in 14–71% of strains within each ST. Thus, there are diverse sources/reservoirs of *C. difficile* that need to be identified. Furthermore, the absence of different lineages within ST2/RT 014/020 strongly suggests diverse sources/reservoirs, more likely within the community [16]. The previous finding of *C. difficile* in smoked and dried fish, traditional foods in Cambodia [17], supports the above argument. *C. difficile* might originate from the community and be brought into healthcare settings or into other communities via humans, animals or food, and vice versa. Investigations of the mechanism(s) behind the transmission of these strains were not performed in this study, but possible transmission routes described in previous studies could be implied [7–9].

Clonal transmission occurring in three genotypes, ST34/RT 056, ST37/RT 017 and ST54/RT 012, might be by chance, but several contributing factors could be postulated, including a higher sporulation rate [18] and higher transmissibility of some strains [19, 20], endemicity of particular genotypes in the region, e.g. ST37/RT 017 [14], the impact of AMR [21] and the possession of different virulence genes [1], as well as the adaptability of each genotype to some yet-to-be-determined local contributing factors [1, 2, 7, 22]. However, clonal strains were not identified between healthcare and community settings; thus, factors limiting the transmission of *C. difficile* within the two settings should be considered, including antimicrobial and other medication consumption [7], endemicity of other infectious diseases [23], living styles or local food composition.

### *C. difficile* strains in Cambodia and Thailand

Comparative genomic analysis showed no clonal strains between the two countries. This does not exclude direct transmission between the two countries as strains were isolated at different times: strains in Cambodia (2022–2023) were isolated 7–11 years later than strains in Thailand (2014–2018). Based on the *C. difficile* molecular clock, *C. difficile* acquires SNPs at a rate of between 0.22 and 1.40 SNPs per genome per year [12]; thus, the two related strains, CAM085 and CAM187 vs B14-21 and MAR272, within ST54 and ST37 from the two countries, could be closely related and clonal, respectively (Supplementary materials S5 and S6).

With the hypothesis that ST37/RT 017 is an Asian strain [4], as well as the close relationship between people of the two countries, it is not surprising that there was clonal *C. difficile* circulating among neighbouring countries within Asia. However, since *C. difficile* is a One Health issue and the connection between the two countries does not rely only on human movement but also on animal and food importation/exportation, comparative genomic analysis between only human strains is inadequate. Rare cases of serious/complicated CDI in humans have been reported in Southeast Asia [5, 24, 25], and further studies into transmission of *C. difficile* within and across the continent are required.

### Antimicrobial resistance

The acquisition of AMR genes has given *C. difficile* the ability to amplify, e.g. ST1/RT 027 [3] and ST37/RT 017 [4]. The emergence of *C. difficile* ST37/RT 017 occurred following the acquisition of *erm(B)* and the introduction of clindamycin in clinical practice. In the current study, none of ST37/RT 017 contained *erm(B)*, but all of them had *tet(M)*, suggesting diverse original reservoirs/sources of ST37/RT 017 in the community rather than in healthcare facilities, though a pair of clonal strains was identified within the healthcare setting (Fig. 2). *C. difficile* ST35/RT 046 was more likely to be present in healthcare settings rather than in the community due to its higher proportion of resistance to antimicrobials than other STs [7, 21]. The reasons why ST35/RT 046 was more resistant to antimicrobials than other STs were not investigated in the current study, but Werner *et al*. [26] and Norén *et al*. [27] suggested that *C. difficile* ST35/RT 046 could be zoonotic; thus, the higher proportion of resistance could be due to the use of antimicrobials in livestock, such as pigs, in Cambodia. And thus, the sources of ST35/RT 046 could be in the community rather than healthcare settings.

The discordance between phenotypic and genotypic resistance in vancomycin, fluoroquinolones, tetracyclines and macrolide–lincosamide–streptogramin B agents points to defects in a gene expression channel and other factors besides the presence of resistance genes. It is well documented that the presence of resistance determinants varies by RT and clade [28]. In the current study, *C. difficile* ST37/RT 017 clade 4 all contained *gyrB* p.S366A, remaining susceptible to moxifloxacin, genotypically not containing *erm* genes but containing *tetM*, suggesting that Cambodian ST37/RT 017 strains might be different from strains from other regions, but also possibly because these ST37/RT 017 strains were from asymptomatic cases, while strains in previous studies were mostly from disease [28]. Nevertheless, the overall findings of AMR determinants in this collection of Cambodian *C. difficile* strains occurred at a higher prevalence compared to collections of strains from around the world between 2000 and 2024: *erm(B*) (41.7% vs 19.09%), *tet(M)* (70.8% vs 18.62%), *blaCDD-2* (58.3% vs 30.32%), but less remarkable for *blaCDD-1* (41.7% vs 69.27%), respectively [1], pointing to possible discrete contributing factors in this country. Noticeably, there was also a significant difference in the presence and absence of different AMR determinants between the two collections, including *catD*, *aadE*, *nimB*, *blaZ*, *rpoB* and *tet(O)*, further suggesting the evolutionary adaptability of *C. difficile* in different environments [1].

Further investigations are warranted to gain insights into these differences and their significance in clinical practice. Interestingly, in the current study, all clade 4 isolates and 67% of isolates in other clades were genetically MDR, almost twice and more than twice the difference, respectively, compared to international collections of *C. difficile* strains, including strains from Asia [28]. These differences indicate a higher burden of AMR in Asia than in other regions, probably due to higher consumption of antimicrobials in Asia, by humans, animals and other agricultural activities.

This study has several limitations. First, all isolates selected for WGS were from asymptomatic infections (or colonization) in both the hospital and community; thus, the discussion on strain relatedness may not represent the actual context of CDI disease in Cambodia, but the spread of *C. difficile*. The genomic comparative analysis between Cambodian and Thai strains was performed between isolates collected at different times, 2022–2023 vs 2014–2018, respectively, contributing to bias in comparison results.

## Conclusions

The current study indicated clonal transmission within healthcare and community settings in Cambodia. However, transmission in these settings was limited. The close relationship between Cambodia and Thailand is a factor that possibly contributes to the spread of *C. difficile* across the border. The five most common toxigenic *C. difficile* genotypes, ST54/RT 012, ST37/RT 017, ST2/RT 014/020, ST35/RT 046 and ST34/RT 056, could originate from both healthcare and community settings, but colonization of individuals in the community before they get admitted to the hospital is quite likely. The identification of reservoirs/sources of *C. difficile* is a crucial step towards further infection prevention and control interventions in the country. There was a poor correlation between phenotypic and genotypic resistance in strains; however, there was a higher proportion of resistance determinants in *C. difficile* identified in Cambodian strains compared to global collections. AMR in *C. difficile* is an area of significant interest in Cambodia, and further investigation is recommended.

## Supplementary material

10.1099/mgen.0.001881 Supplementary Material 1.

## Abbreviations

AMR: antimicrobial resistance
AST: antimicrobial susceptibility testing
CDI: *C. difficile* infection
cgSNPs: core-genome SNPs
gDNA: genomic DNA
MDR: multidrug resistance
OPD: outpatient department
RT: ribotype
RTs: ribotypes
ST: sequence type
WGS: whole-genome sequencing
